# The Role of Spirituality and Religion in Physician and Trainee Wellness

**DOI:** 10.1007/s11606-021-06808-3

**Published:** 2021-06-09

**Authors:** Kristin M. Collier, Cornelius A. James, Sanjay Saint, Joel Howell

**Affiliations:** 1 Department of Internal Medicine, University of Michigan Medical School, Ann Arbor, MI USA; 2 Medical Service, VA Ann Arbor Healthcare System, Ann Arbor, MI USA; 3 Department of History, University of Michigan, Ann Arbor, MI USA

## Abstract

Burnout in medicine is a substantial problem with adverse consequences for both physicians and the patients who they treat. In our efforts to combat burnout, we must consider every tool at our disposal, since a complex problem requires a multifaceted approach. Recognizing that many physicians derive meaning from spirituality and religion, attempts to improve physician and trainee wellness should acknowledge the importance of religion and spirituality for self-care more than has heretofore been the case.

Even before COVID-19, burnout was a problem for medical practitioners.^1^ The pandemic has placed additional stressors on physicians and trainees. Because burnout is associated with suboptimal patient care,^2^ it carries risks not only for physicians but also for their patients. Primary care physicians may be at higher risk for burnout as compared to physicians in other specialties.^3^ Given the prevalence of burnout and the complexity of the root causes, we should consider broadening our approaches to improve physician wellness.^4–7^ Unfortunately, religion and spirituality have been largely absent from discussions about physician and trainee wellness. This may be due, in part, to lack of awareness of their importance. It may also be due to religion and spirituality being seen as in conflict with scientific twenty-first century medicine. But such a conflict is artificial for many people. As we strive to achieve a holistic model of care like the biopsychosocial-spiritual model proposed by Onarecker and Sterling^8^ for our patients and ourselves, we need to take account of the meanings that many physicians derive from spirituality and religion. In this paper, we suggest that attempts to improve physician and trainee wellness should pay greater attention to the importance of religion and spirituality for self-care.

Spirituality relates to a person’s search for meaning and therefore is integral to their self-identity. Religion is one way that people may choose to express meaning. Most physicians are spiritual. A majority of physicians hold religious beliefs, with over half of attending physicians saying that they believe in a God.^6^ Religious beliefs are not only common in attending physicians, but are prevalent in physicians in training as well.^9^ The ability to see meaning in life and at work has been shown to be protective against burnout for physicians, and recommendations to alleviate and prevent healthcare worker burnout have been made around enhancing meaning at work.^10^ Moreover, religious beliefs often guide important personal decisions for many clinicians. For example, almost one-third of physicians chose a career in medicine because of spiritual and/or faith commitments.^6^ Religion plays an essential role in the daily life of many physicians.^11^

To approach the importance of religion and spirituality from a different context, consider how initiatives to promote diversity and inclusion have encouraged the medical profession to appreciate the value of being members of a diverse community. Human beings are complex creatures. Just like all other members of society, healthcare providers’ race and gender are a central part of their identity, both within and outside of the hospital. Consideration of race and gender has become central in efforts to improve the diversity of the healthcare workforce. There are many facets to one’s identity, however. Another overlapping part of that identity includes beliefs about religion and spirituality. There are many important benefits of a diverse medical school climate, one of which is association with improved self-reported mental health of medical students. In one study by Hardeman et al.,^12^ greater exposure to a negative medical school diversity climate among medical students was associated with an increase in self-reported depressive symptoms. To diminish burnout, we must achieve a deeper understanding of the people we wish to help. In so doing, we should acknowledge the importance of religious and spiritual practices for many of our colleagues, just as we would for any other aspect of their identity. This identity helps people find meaning, which is essential in the fight against burnout.

Respecting the importance of religious and spiritual identity can help healthcare leaders do a better job of supporting our colleagues and trainees. Regardless of their own beliefs related to religion and spirituality, attending physicians, program directors, chairs, or deans need to be aware that many people who work in programs that they oversee include religion and spirituality as an essential part of their self-identity. Not only are these beliefs important in our trainees and physician colleagues, but these beliefs are also likely to be very important to other members of our multidisciplinary teams. Unlike other aspects of diversity, one’s religion—and depth of religious commitment—may not be immediately visible to the casual observer. As supervising physicians, do we make it explicit that we realize how important faith is, not only for many of our patients,^13^ but also for many of our colleagues and trainees? Do we recognize the bias that may exist against people with religious traditions of all types? There is an ugly history of religious discrimination in medical school admissions.^14^ Are we as supportive of religious diversity as we are to other facets of diversity? How can we champion and intentionally model an integrated personal-professional identity that makes space for a religious or spiritual commitment for those physicians who choose to do so? Championing our trainees who have faith and/or spiritual commitments may involve allowing trainees to take time for daily prayers, mentoring such trainees, or supporting discussion groups or lecture series on the intersection of health, spirituality, and religion, similar to those offered by the University of Michigan Medical School Program on Health, Spirituality and Religion, the University of Chicago Program on Medicine and Religion, or the Theology, Medicine and Culture Initiative at Duke. Organizational accommodation would include making sure there are communal spaces available for prayer or reflection, or having menus that reflect the dietary needs of those who keep Kosher or who prefer Halal offerings. Having these accommodations would be similar to providing space accommodation for lactating women or providing plant-based menu options for those who avoid eating meat.

There is empirical evidence that religion and spirituality may protect emergency physicians against maladaptive behaviors caused by burnout.^15^ Spirituality may also be consistent with personality traits that protect against burnout. In one study, internal medicine residents who engaged in an active spiritual life were less susceptible to burnout.^16^ Another study demonstrated that extrinsic and intrinsic religiosity predicted psychological well-being in medical students.^17^ Finally, religious service attendance among healthcare workers was found to be associated with a significantly lower risk of a “death from despair”—including suicide.^18^ While these findings could reflect an element of selection bias, the fact that other studies have demonstrated a protective role for religious and spiritual beliefs and practices suggests that there may be a significant protective effect, even though the precise mechanism through which the benefit may be imparted remains unclear. Unfortunately, this part of their humanity is too often ignored.

Spiritual and/or religious beliefs may be particularly important for people in distress. The COVID-19 pandemic has put additional pressure on the physician workforce. The impact of new workflows as a result of the pandemic, the anxiety of seeing colleagues and patients becoming ill, and in some cases dying from the virus has caused significant angst in physicians at every level. And the effects of the pandemic on the physician workforce will not cease once the pandemic is over. The backlog of medical care, the patients that will likely present with delayed diagnoses, and the ongoing financial implications are daunting. Chronic, ongoing stress and moral distress, even pre-pandemic, was a risk factor for burnout^10^ and the pandemic will likely serve to accelerate the burnout crisis. The ultimate consequence of burnout may be suicide. Physicians are at increased risk of death by suicide.^19^ Suicidal ideation is all too common, reported by up to 6% of medical trainees over the past 2 weeks.^20,21^ Religiosity may protect against death by suicide. For example, the Nurses’ Health Cohort study showed religious service attendance was associated with lower risk of suicide in women.^22^ Supporting our colleagues’ faith-based practices may thus help prevent suicide.

We witnessed at our own institution a recent example of how easy it is to overlook the possible connection between spirituality and wellness. After a medical student died by suicide, medical school leadership sent the medical school community a kind, supportive email outlining many useful resources for people who needed help processing this tragedy. However, one potential area for supportive help was noticeably absent: spiritual care. After the omission was pointed out, leadership quickly acknowledged this oversight and sent a new email listing spiritual care as a resource. Our spiritual care department, like most across the country, is helpful to not only patients but also students, faculty, and staff. However, as is often the case, spiritual care may not always be high on the “radar” of those in leadership and therefore has the potential to be omitted when it comes to offering support for resilience and wellness.

If we fail to acknowledge the importance that spirituality and religion hold for many of our colleagues and trainees, we risk marginalizing a core feature of their identity. It may be one that led them to choose medicine as a vocation, protected them against burnout, and provided them with a sense of meaning. Asking people to “leave their faith at the door” when they enter their place of work may not only be inappropriate but may also cause harm as they are then without a core part of their identity, one through which they derive meaning and purpose. For many, putting aside their faith beliefs would be nearly impossible because of the significant impact that their religion and spirituality has on every aspect of their lives, including their work. As we craft systems to confront burnout and enhance wellness, if we do not recognize religion and spirituality as one possible route to maintain wellness, some physicians and trainees may feel like their own spiritual or religious practice does not have a legitimate place in their identity as a physician. In our efforts to combat burnout, we should consider every tool at our disposal. The pandemic has brought the need to be multifaceted in our approach to burnout into sharp acuity. If we want to develop a comprehensive approach to the crisis of burnout, we neglect spirituality at the peril of far too many of our colleagues.
